# Co-expression analysis identifies putative targets for CBP60g and SARD1 regulation

**DOI:** 10.1186/1471-2229-12-216

**Published:** 2012-11-16

**Authors:** William Truman, Jane Glazebrook

**Affiliations:** 1 Department of Plant Biology, Microbial and Plant Genomics Institute, University of Minnesota, 1500 Gortner Avenue, Saint Paul, MN, 55108, USA

**Keywords:** SID2, CBP60g, SARD1, WRKY28, Salicylic acid, Plant immunity

## Abstract

**Background:**

Salicylic acid is a critical signalling component in plant defence responses. In Arabidopsis, isochorismate synthase encoded by *SID2* is essential for the biosynthesis of salicylic acid in response to biotic challenges. Recently, both the calmodulin binding protein CBP60g and its closest homolog, the non-calmodulin binding SARD1, have been shown to bind to the promoter region of *SID2*. Loss of both *CBP60g* and *SARD1* severely impacts the plants ability to produce SA in response to bacterial inoculation and renders the plant susceptible to infection. In an electrophoretic mobility shift assay CBP60g and SARD1 were shown to bind specifically to a 10mer oligonucleotide with the sequence GAAATTTTGG.

**Results:**

Gene expression profiling on a custom microarray identified a set of genes, like *SID2*, down-regulated in *cbp60g sard1* mutant plants. Co-expression analysis across a defined set of ATH1 full genome microarray experiments expanded this gene set; clustering analysis was then applied to group densely interconnected genes. A stringent threshold for co-expression identified two related calmodulin-like genes tightly associated with *SID2*. *SID2* was found to cluster with genes whose promoter regions were significantly enriched with GAAATT motifs. Genes clustering with *SID2* were found to be down-regulated in the *cbp60g sard1* double mutant. Representative genes from other clusters enriched with the GAAATT motif were found to be variously down-regulated, unchanged or up-regulated in the double mutant. A previously characterised co-expression between *SID2* and *WRKY28* was not reproduced in this analysis but was contained within a subset of the experiments where *SID2* was co-expressed with *CBP60g* or *SARD1*.

**Conclusion:**

Putative components of the CBP60g SARD1 signalling network have been uncovered by co-expression analysis. In addition to genes whose regulation is similar to that of *SID2* some are repressed by CBP60g and SARD1.

## Background

Two principal mechanisms, with overlapping components, exist to protect plants from infection. Pattern triggered immunity (PTI) involves the recognition of conserved, indispensible microbial structures, such as flagellin. These Microbe Associated Molecular Patterns (MAMPs) are recognised by Pattern Recognition Receptors (PRRs) such as FLS2, which recognizes flagellin, and stimulate a signalling network to elaborate an appropriate defence. In order to break free of this basal immune response adapted pathogens must produce and deliver effectors capable of disarming the plants surveillance and countermeasures. The second mechanism of protection therefore is Effector Triggered Immunity (ETI) whereby plants monitor effectors or their targets with Resistance (R) gene products. Recognition of pathogen effectors again stimulates a signalling network with many elements common to that of PTI but with typically more drastic consequences [1]. Plant hormones play critical roles in the signalling following both PTI and ETI, with salicylic acid (SA) central in mediating protection against biotrophic and hemi-biotrophic pathogens [2,3]. Salicylic acid accumulates both locally and systemically following infection and is essential for establishment of Systemic Acquired Resistance (SAR) and the development of durable, broad spectrum resistance against normally virulent pathogens [4,5].

For both ETI and PTI the accumulation and action of SA is dependent on several shared components. In *Arabidopsis thaliana* the EDS1/PAD4 node lies upstream of SA biosynthesis, as the two interacting proteins are essential for activation of the SA signalling sector [6,7]. SID2 has been identified as a critical component in the biosynthesis of SA in response to biotic challenge; *SID2* encodes an isochorismate synthase capable of catalysing the formation of the SA precursor isochorismate from chorismate [8]. Also critical for the accumulation of SA is the MATE transporter EDS5, which may be involved in the transport of a biosynthetic precursor of SA [9]. Downstream of SA biosynthesis, NPR1 is involved in the activation of SA-dependent gene expression. Suitably high SA levels and appropriate redox conditions result in NPR1 monomerisation allowing it to enter the nucleus and interact with transcription factors of the TGA family [10-12]. Recent studies have identified either NPR1 or the paralogs NPR3 and NPR4 as SA receptors [13,14]. Wu *et al*. showed that the interaction of SA and NPR1 produced a conformational change allowing the NPR1 BTB/POZ domain to interact with TGA2. While Fu *et al*. did not observe SA binding by NPR1 they showed that NPR3 and NPR4 could act as SA-dependent adapters for the proteasomal degradation of NPR1 whose different affinity for SA subtly modulates the response to different SA concentrations.

With SID2 occupying a critical role in the transduction of defence signalling through SA, much effort has been made to understand its regulation. Positive regulators of *SID2* expression have been identified, such as WRKY28 which has been shown to bind to the *SID2* promoter and induce *SID2* expression in transfection assays. Electrophoretic mobility shift assays (EMSA) revealed that WRKY28 bound to a modified version of the consensus W-box motif that retains the TGAC core [15]. Negative regulators of *SID2* expression have also been uncovered; EIN3 has been shown to bind to the *SID2* promoter and combined mutations of *ein3* and its close homolog *eil1* showed elevated *SID2* expression, SA accumulation and increased resistance to bacterial infection [16]. Similarly, three related NAC transcription factors (ANAC019, ANAC055 and ANAC072) were found to inhibit *SID2* expression, SA accumulation and resistance to bacterial infection with ANAC019 shown to bind to the *SID2* promoter [17].

Two further genes involved in the regulation of *SID2* are *CBP60g* and *SARD1*. CBP60g is a member of a family of calmodulin (CaM) binding proteins that was identified as being strongly induced in response to MAMPs treatment. Plants carrying *cbp60g* null mutations were compromised in the induction of *SID2* and accumulation of SA [18]. CBP60g was shown to bind CaM in a Ca^2+^ dependent fashion; *cbp60g* transgenes with mutations in the CaM binding domain that abolished the CaM interaction were incapable of complementing the null mutant. Independently, the closest homolog of CBP60g was identified in a screen for mutants defective in systemic acquired resistance and named SARD1 [19]. SARD1, while more closely related to CBP60g than the rest of the CBP60 family, does not bind CaM [19,20]. Both *cbp60g* and *sard1* were impaired in SAR with the double mutant more strongly affected [19]. Lines over-expressing *SARD1* accumulated more SA than wildtype plants [19] while the double knockout mutant was severely compromised in SA accumulation in response to infection [19,20]. Both CBP60g and SARD1 were shown to bind to the promoter of *SID2* and in EMSA experiments a central DNA binding domain of both proteins was found to bind to an oligomer with the sequence GAAATTTTGG selected from the *SID2* promoter [19]. *CBP60g* and *SARD1* have partially redundant function in SA signalling with both mutants affecting SA accumulation and pathogen growth but the double mutant exhibiting a greater than additive effect [19,20]. While clearly overlapping in function there are a variety of distinctions in addition to the requirement of CaM binding: CBP60g appears to have more influence over the early events in defence signalling with SARD1 playing a more prominent role later; MAMPs triggered signalling is more greatly affected by the loss of CBP60g than the loss of SARD1 [20]. At the transcriptomic level the expression fingerprint of *cbp60g* more closely resembles that of *sid2* than *sard1* does during MAMPs responses while the trend was reversed later time-points with virulent bacterial infection [20].

While the *cbp60g sard1* mutant drastically reduces *SID2* expression and SA accumulation upon biotic challenge the double mutant is more susceptible to *Pseudomonas syringae* pv *maculicola* ES4326 (*Pma* ES4326) than *sid2-2* indicating a role for CBP60g and SARD1 in SA-independent defence signalling [20]. Potential targets for CBP60g SARD1 regulation were identified using a custom microarray where 25 genes (from an array of 571 genes) including *SID2* were down-regulated in the double mutant. Analysis of the promoters of these genes found a significant enrichment of a GAAATTT motif, a fragment of the oligomer used in Zhang *et al*.’s EMSA study. Similarly Zhang *et a*l. noted an enrichment of AATTTT motifs in genes up-regulated in other PTI and ETI studies.

With the advent of whole genome transcriptional profiling many studies have made use of the abundance of microarray data to identify new pathway components based on their co-expression with known elements. For example, additional enzymes involved in cellulose synthesis and flavonoid biosynthesis were uncovered based on their correlation with known genes across publicly available array data [21,22]. This type of analysis has also uncovered new regulatory elements controlling glucosinolate biosynthesis and fatty acid biosynthesis [23,24]. In fact it was co-expression analysis that first uncovered the regulation of *SID2* by WRKY28 [25]. Interestingly, the correlation between *SID2* and *WRKY28* was only observed when restricted to a subset of array experiments involving stress treatments. Given the enrichment of putative CBP60g SARD1 motifs within the sample of genes down-regulated in *cbp60g sard1* plants we decided to use co-expression analysis to expand this subset and search for similar motif enrichment in order to identify additional potential targets for CBP60g SARD1 control and additional components of the signalling network.

## Results

### Expression patterns of *CBP60g*, *SARD1*, and *SID2* are correlated

**Figure 1:**
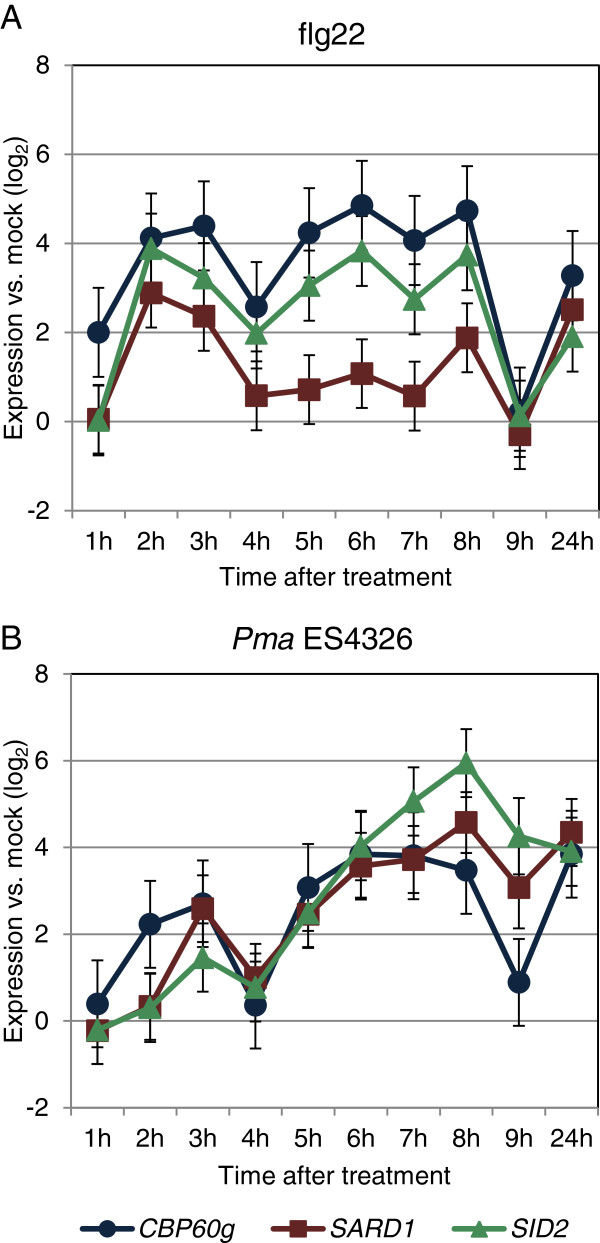
***CBP60g*, *SARD1 *and *SID2 *are co-expressed in response to MAMPs treatment and *Pma *ES4326 infection.** Wildtype Col-0 Arabidopsis plants were treated with 1 μM flg22, *Pma* ES4326 (OD_600_ = 0.01) or mock treated with water. qPCR data from two biological replicates were combined using a mixed linear model and the expression relative to *Actin2* calculated. The log_2_ ratio of treatment relative to mock is plotted with error bars representing the standard error of the difference between means. (**A**) With flg22 the Pearson correlation coefficient for *CBP60g:SID2* was 0.92 and 0.66 for *SARD1*:*SID2.* (**B**) Following *Pma* ES4326 inoculation the Pearson correlation coefficient values are 0.64 for *CBP60g:SID2* and 0.93 for *SARD1:SID2.*

Our previous work suggested that *CBP60g* plays a greater role than *SARD1* during a MAMP response, while the reverse is true during the response to *Pma* ES4326. To test for a similar effect in the relationship of the expression levels of *CBP60g* and *SARD1* to that of *SID2*, we monitored expression of these three genes following infiltration of leaves with the flagellin fragment flg22 or *Pma* ES4326, sampling every hour for the first 9 h following infection followed by a final sampling at 24 h. Figure 1 shows that following flg22 treatment, CBP60g induction precedes *SARD1* induction, with expression of *CBP60g* already significantly up-regulated at 1 h, and maintaining a stronger fold-induction throughout the time course. The expression profile of *CBP60g* is closely mirrored by that of *SID2* with the slight difference that CBP60g induction is more rapidly activated. Accordingly, the Pearson’s correlation for the similarity between the *CBP60g* and *SID2* expression patterns was high (0.92) compared to the *SARD1*:*SID2* correlation (0.66). A different pattern was observed following inoculation with *Pma* ES4326. While *CBP60g* is again faster to respond to infection, from 3h *SARD1* up-regulation matches that of *CBP60g* and then exceeds it from 8h onwards. The later elevation of *SARD1* transcription more closely matches that of *SID2* than *CBP60g*. The correlation values are reversed relative to flg22 inoculation, at 0.64 for *CBP60g:SID2* and 0.93 for *SARD1:SID2*. These results are consistent with the idea that *CBP60g* has a greater effect than *SARD1* on *SID2* expression using a MAMPs response, while the reverse is true during response to *Pma* ES4326.

### Selection of data sets for defining the *CBP60g/SARD1* regulon

**Figure 2:**
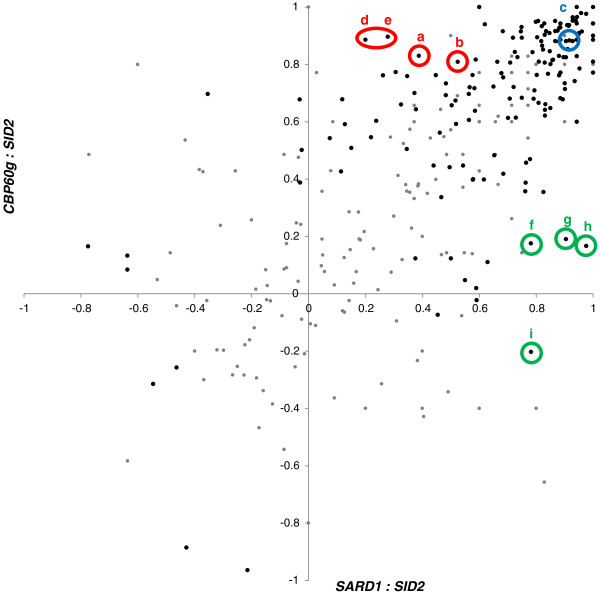
**Significant *SID2:CBP60g/SARD1 *correlation is observed in numerous publicly available microarray datasets.** Affymetrix ATH1 array experiments were RMA normalised and the Spearman rank correlation coefficient calculated between probesets representing *SID2* (262177_at) and *CBP60g* (246821_at) and *SARD1* (260046_at) plotted. Darker points represent experiments where at least one correlation has a p-value no greater than 0.05. Highlighted experiments represent: (**a**-**c**) assorted MAMPs treatments; (**d**-**e**) plastid function; (**f**) oomycete infection; (**g**) SA and ABA signalling mutants; (**h**) exocyst component mutants; (**i**) potassium starvation.

We hypothesized that genes under the control of CBP60g and SARD1 could be identified by mining public gene expression data for genes co-expressed with the 45 genes whose expression was found to be suppressed in *cbp60g*, *sard1* or *cbp60g sard1* mutant plants using a small defence-related custom microarray (GEO: GSE18865 [20]). To do this, we wanted to select an appropriate set of data, as inclusion of many experiments in which defence genes are not induced is likely to increase the noise in the analysis. For example, the relationship between WRKY28 and SID2 was only observed in a subset of the ATTEDII co-expression database restricted to stress-associated profiling experiments and excluding other data [25]. We collected data from 308 expression profiling experiments conducted using the Affymetrix ATH1 Arabidopsis array, with treatments related to pathogen infection, stress responses, hormone treatment or associated mutations, and developmental series. The experiments ranged in size from six to several hundred arrays. Data from each experiment were processed identically; a conservative approach was taken to quality control with any outlying arrays being removed prior to batch normalization and summarization by RMA. In each experiment, the Spearman rank correlation coefficients for *CBP60g:SID2* and *SARD1:SID2* were calculated along with the associated p-value for significance of correlation. The results are shown in Figure 2. In many experiments, both correlations are similarly strong, but there are also experiments in which only *CBP60g* or only *SARD1* is strongly correlated with *SID2.* In several experiments where a MAMPs elicitor is applied the correlation between *CBP60g* and *SID2* is greater than the correlation between *SARD1* and *SID2*. For example in the experiment marked (a) (NASCARRAYS122) seedlings were treated with flg22, the bacterial protein HrpZ, the fungal protein NPP1 or a preparation of bacterial lipopolysacharrides, the *CBP60g* correlation is 0.83 while the *SARD1* correlation is 0.39. Similarly in (b) (Ausubel lab IMDS – Flg22 and OGs [26]) with treatments of flg22 and the plant-derived danger signal oligogalacturonides the *CBP60g* correlation is 0.81 while the SARD1 correlation is 0.52. However, not all MAMP treatment experiments gave rise to the same discrimination, in (c) (Ausubel lab IMDS – Chitin 8mer) treatment with fungal-derived chitin fragments produce equally strong correlations for both *CBP60g* and *SARD1*, 0.88 and 0.90 respectively. Other experiments which exhibited stronger *CBP60g SID2* correlation included experiments investigating plastid function. In (d) (EBI Arrayexpress E-MEXP-2927 [27]) null mutations in the plastid biogenesis component SCO3 produce a *CBP60g* correlation of 0.89 while the *SARD1* correlation was 0.2. The impact of excess light or application of an electron transport inhibitor in (e) (EBI Arrayexpress E-MTAB-403) also resulted in a specific *CBP60g:SID2* correlation, 0.90 compared with 0.28 for *SARD1*. Array experiments where a stronger *SARD1*:*SID2* correlation was observed included: (f) *Phytophthora parasitica* infection of roots; (g) combined mutations in ABA signalling and SA biosynthesis; (h) mutations in the exocycst component EXO70A1; (i) comparisons of potassium starvation and caesium toxicity (NASCARRAYS468; GEO GSE16913, [28]; NASCARRAYS435; NASCARRAYS105). While there are interesting trends in the specific, discriminatory associations between *CBP60g* or *SARD1* and *SID2* there are not currently sufficient datasets to construct specific co-expression networks. In the majority of experiments where *CBP60g* or *SARD1* was strongly and significantly correlated with *SID2* the homolog was also positively correlated, providing a sufficiently large dataset to form robust co-expression networks. Based on these results, we decided to use the 125 experiments, comprising 2,245 arrays, in which either *CBP60g* or *SARD1* showed a strong and significant correlation with *SID2* of at least 0.7 and a p-value of no more than 0.05.

### Some clusters of genes co-expressed with *CBP60g/SARD1*-dependent genes have promoters enriched with GAAATT motifs

We reasoned that by clustering genes with expression patterns similar to the 45 genes that showed reduced expression in *cbp60g*, *sard1*, or *cbp60g sard1* plants, we might identify additional defence genes whose expression levels are controlled by CBP60g and/or SARD1. Prior to clustering, we explored different criteria for selection of co-expressed genes. We used Spearman’s rank correlation as a measure of similarity, and determined the number of probesets obtained using different cut-off values from 0.85 to 0.70 (Table 1). For each set of genes obtained, we counted the numbers of GAAATT motifs in their promoters. Previous promoter analysis of differentially expressed genes had scored GAAATTT as the most significant sub-component of the CBP60g SARD1 binding motif [20]. However, in pilot clustering analysis and in all subsequent analyses the shortened GAAATT motif was found to have greater statistical over-representation in clusters containing *SID2* (Additional file 1: Table S1). Hence we used the GAAATT motif rather than the longer GAAATTT motif to evaluate the various thresholds and clustering metrics for analysis. The 0.85 correlation cut-off was clearly too stringent, as it identified 59 probesets, only 14 more than the 45 used to begin the analysis. The 0.80 cut-off identified 128 probesets, with 2.77 motifs per gene. This seemed a reasonably stringent criterion, and we used these 128 probesets for one clustering analysis - experiment #1. When the correlation cut-off was relaxed to 0.70, 518 probesets were identified, albeit with a lower average density of motifs per gene of 2.52. However, these included many corresponding to genes with 4 or more motifs in their promoters, suggesting that the 0.80 cut-off may exclude some potential targets for CBP60g SARD1 regulation. We used this larger set for a second clustering analysis - experiment #2.

**Table 1:** Assessing different correlation thresholds in forming a co-expression network

**Gene correlation cut off (Spearman rank correlation)**	**0.85**	**0.8**	**0.75**	**0.7**
Number of probesets	59	128	275	518
Mean number of GAAATT motifs per gene	2.55	2.77	2.68	2.52
Genes with more than 3 GAAATT motifs	19	45	90	145
Genes with more than 4 GAAATT motifs	8	22	45	72
Genes with more than 5 GAAATT motifs	5	17	24	38
Genes with more than 6 GAAATT motifs	3	8	10	17
Genes with more than 7 GAAATT motifs		3	4	7

For both clustering experiments, we used DPClus [29] to identify densely interconnected nodes within the co-expression network with connections being defined as correlations of 0.8 or greater for experiment #1 and 0.7 or greater for experiment #2. The generation of clusters was constrained by a threshold for the minimum density of connections within a cluster and a parameter controlling the periphery tracking of clusters such that sparsely connected nodes would be ejected from a cluster even if the average connection density passed the threshold. These two parameters affect the clustering resolution and the capacity to distinguish sub-networks within the co-expression network. Overlapping clusters could be formed permitting one gene node to span multiple clusters while some sparsely connected genes could be excluded from all clusters. Various parameters were tested and the cluster or clusters containing *SID2* evaluated. In each instance the GAAATT motif was significantly over-represented in the *SID2* cluster and parameters that gave rise to the maximum statistical significance observed were chosen for the final analysis – a minimum density value of 0.75 and a CP threshold of 0.75.

**Figure 3:**
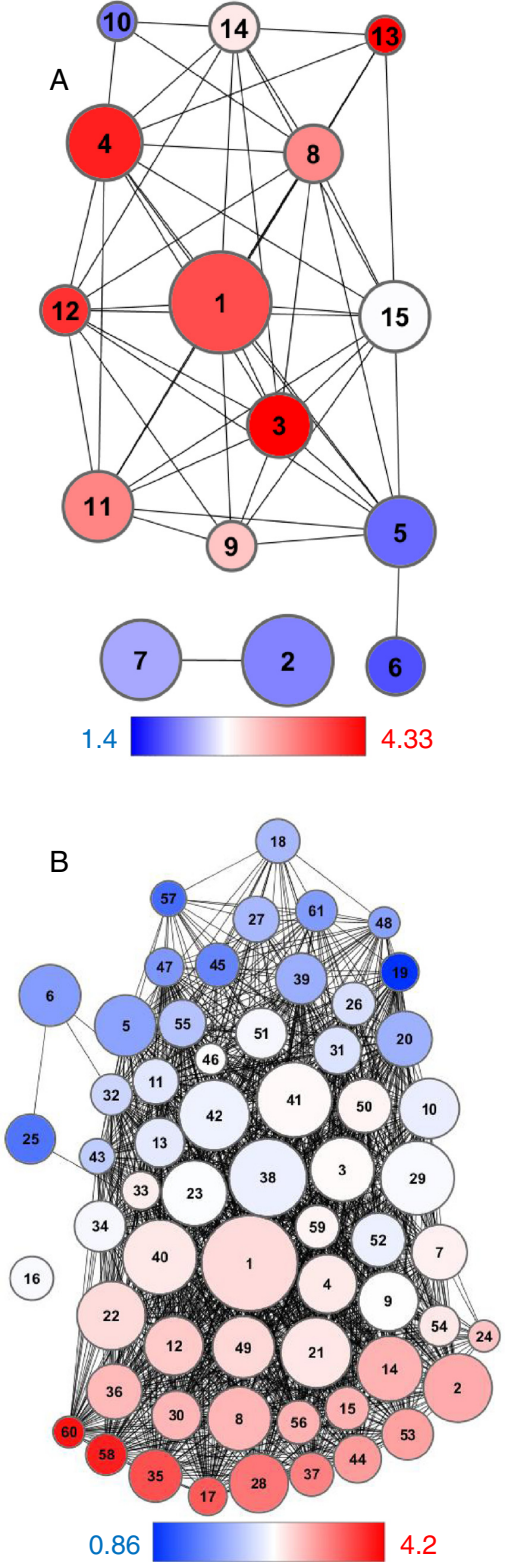
**Clusters of genes with GAAATT enriched promoters identified within the *CBP60g / SARD1 *co-expression network.** 45 genes down-regulated in *cbp60g*, *sard1* or *cbp60g sard1* mutants were used to seed a co-expression network. (**A**) In experiment #1 a Spearman rank correlation coefficient threshold of 0.8 defined a network of 128 probesets. DPClus formed 15 overlapping clusters. (**B**) In experiment #2 a correlation threshold of 0.7 defined a network of 518 probesets which were organised into 61 overlapping clusters. Circles represent clusters of genes and edges represent correlation between members of different clusters. The number of genes in a cluster is proportionate to the size of the circle. The colour of the cluster reflects the average number of GAAATT motifs within the 1500 bp promoter region of cluster members, red indicates an over-representation, blue under-representation and white the genome average.

Figure 3a shows the result of experiment #1. The 128 probesets formed 15 clusters, ranging in size from 3 to 16 genes, with average numbers of motifs per gene ranging from 1.4 to 4.33 motifs per gene. Figure 3b shows the result of experiment #2. The 518 probesets formed 58 clusters, ranging in size from 5 to 113 genes, with average numbers of motifs per gene ranging from 0.86 to 4.2 motifs per gene (Additional file 2: Table S2 and Additional file 3: Table S3).

### The *SID2* clusters are enriched for GAAATT motifs and defence genes

**Figure 4:**
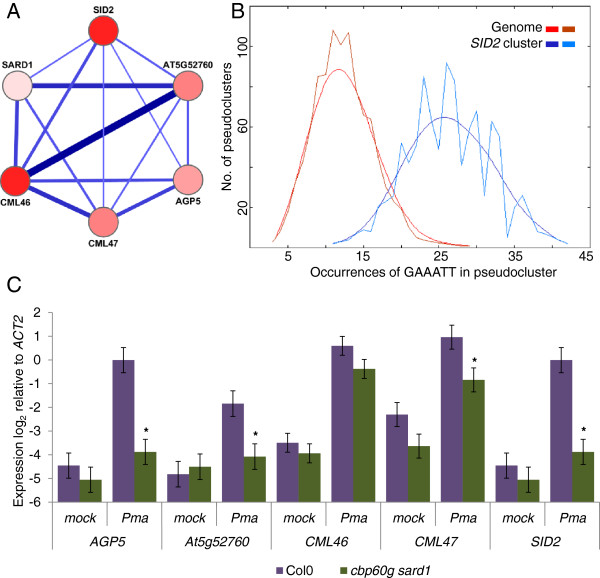
***SID2 *is tightly co-expressed with *SARD1 *and calmodulin-like genes with GAAATT abundant promoters.** (**A**) The internal structure of cluster 3 from experiment #1. Edges between genes show correlation above the threshold of 0.8 with thickness proportional to the magnitude of correlation. Genes are coloured according to the number of GAAATT motifs present in the 1500 bp promoter region from white for genes with 0 motifs to red for a maximum of 7. (**B**) POBO analysis of GAAATT motif frequency in the 1500 bp upstream of transcription start sites. 1000 pseudoclusters of six genes were generated both from within cluster 3 and the genome background; jagged lines show the motif frequencies from which a fitted curve was derived. GAAATT motifs were found to be significantly over-represented with a p-value < 0.0001. (**C**) qRT-PCR measurement of gene expression 24 hpi *Pma* ES4326 inoculation (OD_600_ =0.01). Data from four or five biological replicates were merged using a mixed linear model and the mean log_2_ ratio to *Actin2* expression plotted along with the standard error. Asterisks denote a significant differential expression between wildtype and the *cbp60g sard1* mutant with p-value ≤ 0.05 from a two-tailed *t*-test.

As *SID2* has been reported to be a direct target of CBP60g and SARD1, we studied the clusters containing *SID2* more closely. In Experiment #1, *SID2* is in cluster 3. The internal structure of this cluster is shown in Figure 4a. It includes five genes in addition to *SID2*: The density of connections for the cluster is 1 and the gene most strongly co-expressed with *SID2* is *CML46*. *CML46* and *CML47* are two members of a gene family encoding calmodulin-like proteins containing calcium binding EF-hand domains. They are phylogenetically closely related to a third family member not represented on the ATH1 array, *CML45*[30]. *SARD1* is contained in this cluster (though it is only represented by the more reliable of its two probesets) alongside a putative heavy-metal transporter and *AGP5*. Of the five genes linked to *SID2* only *AGP5* was present on the custom array used to seed this analysis. As shown in Figure 4b, analysis of the GAAATT motif in the promoters of these genes by POBO shows that enrichment of this motif is highly significant (p-value < 0.0001) [31]. qPCR confirmed that expression of all cluster members was induced in response to infection with *Pma* ES4326, this induction was significantly suppressed in the *cbp60g sard1* mutant for *AGP5*, *At5g52760* and *CML47*; *CML46* expression was lower in the double mutant but with a high p-value of 0.097 (Figure 4c). Intriguingly, *CML45*, the nearest homolog of *CML46* and *CML47,* is up-regulated in response to *Pma* ES4326 and this induction is significantly enhanced in *cbp60g sard1* plants (Additional file 4: Figure S1).

**Figure 5:**
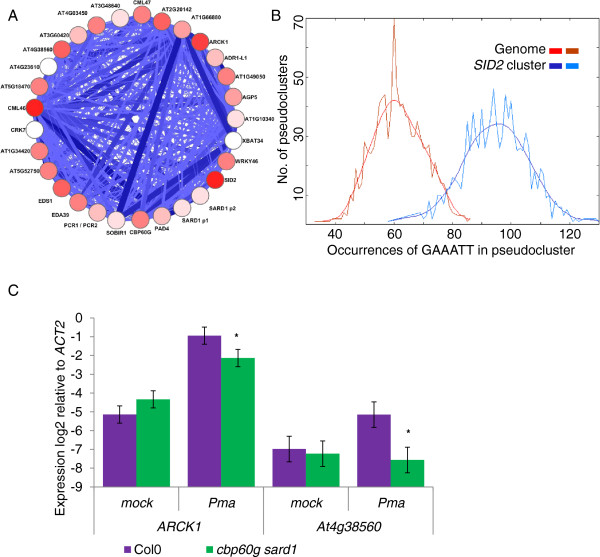
**An expanded SID2 regulon contains *CBP60g*, *SARD1 *and a variety of defence genes with GAAATT rich promoters.** (**A**) The internal structure of cluster 2 from experiment #2. Edges between genes show correlation above the threshold of 0.7 with thickness proportional to the magnitude of correlation. Genes are coloured according to the number of GAAATT motifs present in the 1500 bp promoter region from white for genes with 0 motifs to red for a maximum of 7. (**B**) POBO analysis of GAAATT motif frequency in the 1500 bp upstream of transcription start sites. 1000 pseudoclusters of 30 genes were generated both from within cluster 2 and the genome background; jagged lines show the motif frequencies from which a fitted curve was derived. GAAATT motifs were found to be significantly over-represented with a p-value < 0.0001. (**C**) qRT-PCR measurement of gene expression 24 hpi *Pma* ES4326 inoculation (OD_600_ =0.01). Data from five biological replicates were merged using a mixed linear model and the mean log_2_ ratio to *Actin2* expression plotted along with the standard error. Asterisks denote significant differential expression between wildtype and the *cbp60g sard1* mutant with p-value ≤ 0.05 from a two-tailed *t*-test.

In the expanded clustering of experiment #2 *SID2* is also present in only one cluster – cluster 2. Figure 5a shows that the *SID2* cluster from experiment #2 contains all the genes from the more stringent analysis but is much larger, comprising 31 probesets. The POBO analysis shown in Figure 5b indicates that the enrichment of the GAAATT motif remains highly significant in this cluster. *SID2* and *CML46* are the cluster members with the highest GAAATT motif density with 7 motifs while *ARCK1*, a receptor-like kinase involved in suppressing ABA responses has 6 [32]. qPCR was used to investigate the impact of the *cbp60g sard1* double mutation on two of the genes with GAAATT rich promoters not previously identified as being involved in defence, ARCK1 and the phospholipase At4g38560 (Figure 5c). In addition to both *SARD1* probesets and *CBP60g*, this cluster also includes many genes known to play important roles in plant defence because loss-of-function mutations compromise resistance. These include *PAD4*[33], *EDS1*[34], *ADR1-L1*[35], *SOBIR1*[36] and *WRKY46*[15]. The cluster was significantly enriched for genes with known or putative receptor kinase function and genes implicated in calcium signalling when compared to all the probesets on the array using the Mapman over-representation tool (http://mapman.mpimp-golm.mpg.de/general/ora/ora.shtml, [37]). The heavy-metal transporter gene from the experiment #1 *SID2* cluster was joined by a neighbouring close homolog. Three genes in the cluster were predicted to contain ankyrin repeats.

All 7 members of the cluster tested by qPCR were found to be up-regulated in response to bacterial infection in a *cbp60g sard1* dependent fashion except *CML46* (Figure 4c and 5c). While some cluster members may prove to be targets for CBP60g SARD1 regulation, genetic analysis and transcriptome profiling has placed other components (*PAD4*, *EDS1*) upstream of *CBP60g* and *SARD1* in the immune signalling pathway, thus the co-expression network reveals multiple stages in the control of SA mediated defence signalling. Both *EDS1* and *PAD4* have previously been characterised as SA inducible and their roles in the feed-forward loop amplifying SA signals may best explain their co-expression with *SID2*[38,39].

**Figure 6:**
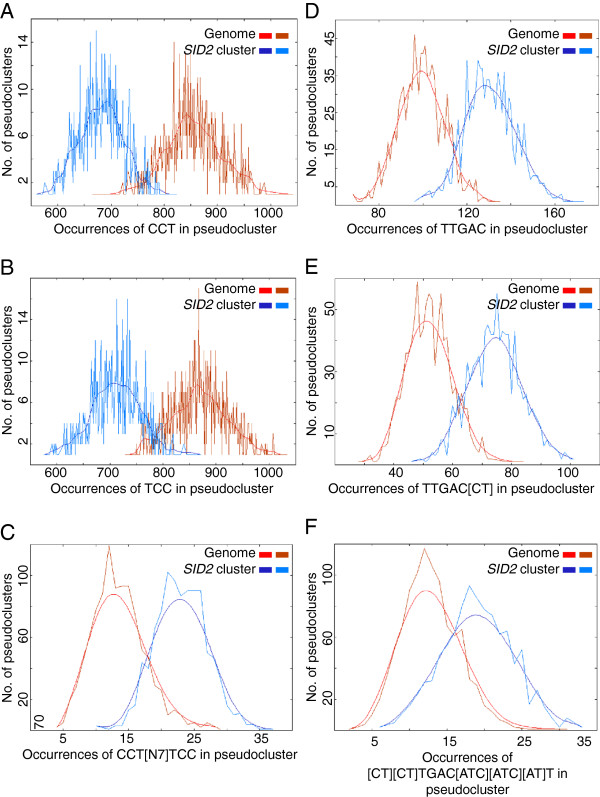
**The *SID2 *regulon is enriched with known cis-elements and novel motifs.** POBO analysis of motif distribution in 1500 bp promoters from experiment #2 cluster 2. 1000 pseudoclusters of 30 genes were generated both from within cluster 2 and the genome background; jagged lines show the motif frequencies from which a fitted curve was derived. (**A**-**C**) CCTNNNNNNNTCC motifs are significantly over-represented with a p-value < 0.0001 though CCT and TCC motifs are significantly under-represented in the cluster. (**D**-**E**) Different versions of the W-box consensus sequence binding site for WRKY transcription factors and (**F**) the defined WRKY28 binding site are significantly over-represented in cluster 2.

Other motifs were observed to be over-represented within this cluster in addition to GAAATT. A CCT n7 TCC dyad was over-abundant while the CCT or TCC submotifs or the dyad with any intervening length other than 7 were all under-represented (Figure 6a-c). This motif was found in the *SARD1* promoter close to the transcription start site. 17 of the 23 CCT n7 TCC motifs present in the cluster fall in the proximal 750 bp of the 1500 bp promoter, a significant bias when compared with the genome distribution by Fisher’s exact test (p-value=0.005) (Additional file 5: Figure S2). No such bias was observed for GAAATT position or strand in any cluster. Various permutations of the consensus WRKY transcription factor binding site W-box and the W-like box described in [15] were also observed to be enriched in the SID2 cluster (Figure 6d-f).

### Genes in other clusters enriched for GAAATT motifs are suppressed, induced or unaffected by the *cbp60g sard1* mutant

**Figure 7:**
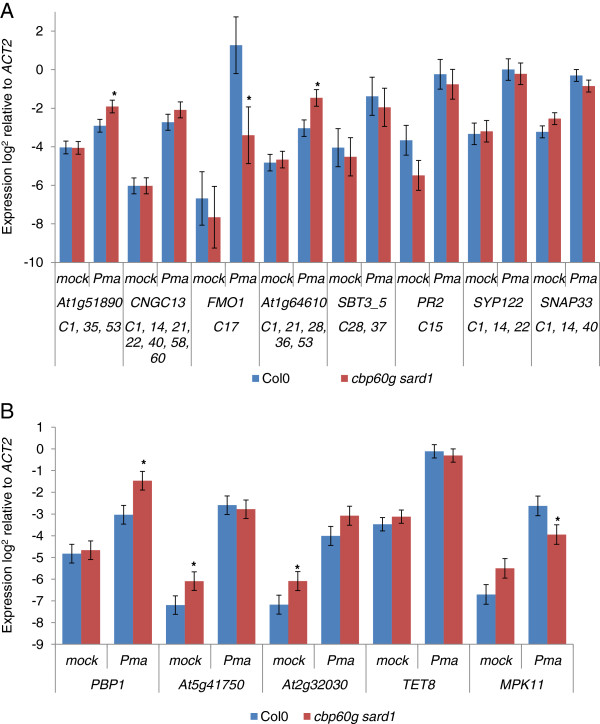
**Putative targets for CBP60g / SARD1 regulation are repressed in *cbp60g sard1 *plants.** Expression of candidate genes with promoters containing multiple GAAATT motifs from the 14 clusters, other than cluster 2, most significantly enriched for GAAATT motifs in experiment #2. (**A**) Candidates from clusters 1, 14, 15, 17, 21, 22, 28, 35, 36, 37, 40, 53, 58, 60. (**B**) Candidates from cluster 8. qRT-PCR measurement of gene expression 24 hpi *Pma* ES4326 inoculation (OD_600_ =0.01). Data from five biological replicates were merged using a mixed linear model and the mean log_2_ ratio to *Actin2* expression plotted along with the standard error. Asterisks denote a significant differential expression between wildtype and the *cbp60g sard1* mutant with p-value ≤ 0.05 from a two-tailed *t*-test.

Expanding the analysis to other clusters with a significant over-representation of the GAAATT motif (Additional file 6: Table S4) uniformly identified pathogen inducible gene expression (Figures 7a and b). However, while some representative (GAAATT abundant) genes from GAAATT-rich clusters, for example cluster 17, were demonstrated to be *cbp60g sard1* dependent for their full induction by qPCR (Figure 7a) or already known to be so from the custom microarray study (*PBS3*, *AIG1*) other GAAATT rich cluster members, for example *CNGC13* from cluster 60, were found to be induced in a *cbp60g sard1* independent fashion. Since GAAATT is a commonly occurring motif in Arabidopsis promoters small clusters when repeatedly sampled with replacement by POBO may be unduly skewed by the presence of one or two genes with a large number of motifs.

One larger cluster, cluster 14 with 26 genes enriched with GAAATT motifs, was found to contain *CBP60g* alongside multiple vesicle trafficking components, including some shown to be important for SA homeostasis [40]. However, no *cbp60g sard1* dependent expression was observed for *SYP122* or *SNAP33* (Figure 7a). W-boxes and another motif, AAGTC, were both observed with significant over-representation in this cluster and may better explain potential co-regulation within the cluster (Additional file 6: Table S4). Another cluster representing genes of clearly linked function was cluster 15, containing the pathogen responsive genes *PR1*, *PR2*, *PR5* and *PNP-A*. However, while *PR1* has previously been shown to require CBP60g and SARD1 for full induction, *PR2* was not significantly affected.

In some instances the GAAATT rich gene selected to monitor a given cluster was found to be up-regulated in the *cbp60g sard1* double mutant relative to wildtype e.g. *At1g51890* and *At1g64610*. This phenomenon was observed in several members of cluster 12 with *PBP1* having a significantly enhanced response to infection in the double mutant while *At5g41750* and *At2g32030* were up-regulated in the mock inoculated mutants. Intriguingly for *MPK11*, which encodes a MAP kinase activated during PTI [41], both a significantly suppressed pathogen response and elevated basal expression were observed in the mutant.

### *WRKY28:SID2* co-expression occurs in a subset of the conditions included in this analysis

**Figure 8:**
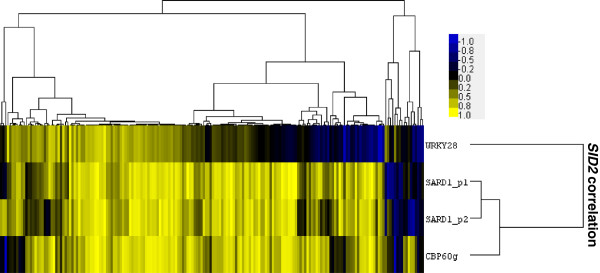
***WRKY28:SID2 *co-expression is restricted to a subset of conditions engendering *CBP60g/SARD1:SID2 *co-expression.** Spearman rank correlation coefficient between *SID2* and *CBP60g, SARD1* and *WRKY28* plotted for the 182 microarray experiments where at least one correlation was significant (p-value ≤ 0.05)*.* Correlation values were sorted by a self-organising maps algorithm and subjected to complete linkage hierarchical clustering. Positive correlations are yellow while negative correlations are blue.

Prior co-expression analysis of biotic stress microarray data had uncovered the regulation of *SID2* by *WRKY28* and the regulation of SA biosynthesis component *PBS3* by *WRKY46*[25]. While *WRKY46* clustered with *SID2*, the proposed downstream target for WRKY46, *PBS3* does not co-cluster with *WRKY46*. Similarly, *WRKY28* was not found to be robustly co-expressed with *SID2* in this study and was not included in the 518 genes selected for analysis in experiment #2. In the selected dataset of 2245 arrays *WRKY28* ranks as the1775^th^ most closely correlated gene with *SID2* with a Spearman rank coefficient of 0.34. The modified W-box motif identified through EMSA as the binding site for WRKY28 is enriched in cluster 2 but weakly in comparison with other motifs investigated (Figure 6f). To identify conditions responsible for the previously reported *WRKY28:SID2* co-expression, microarray studies with a significant *WRKY28:SID2* correlation were plotted alongside *CBP60g:SID2* and *SARD1:SID2* correlations in a heatmap (Figure 8). The association of *WRKY28* and *SID2* comprised a subset of the studies with significant *CBP60g* or *SARD1* co-expression. However, analysis of the 31 experiments comprising 386 arrays where *WRKY28:SID2* expression is significant and greater than 0.7 still resulted in a stronger association between *CBP60g* or *SARD1* and *SID2* than *WRKY28* and *SID2* with *WRKY28* only rising to become the 69^th^ gene most closely correlated with *SID2*.

## Discussion

The various strands of evidence pointing to the discriminatory action of the partially redundant CBP60g and SARD1 in mediating different aspects of the immune response were reinforced when surveying the microarray datasets (Figure 2). As with the flg22 timecourse (Figure 1) a closer association of *CBP60g* and *SID2* was observed in several MAMPs treatment studies and intriguingly, several experiments unrelated to defence. Unfortunately, despite the abundance and variety of microarray experiments publicly available there are currently too few to build independent correlation networks for *CBP60g* and *SARD1*. Aoki *et al*. reported that a minimum of 100 arrays is required for stability in the density of gene co-expression networks [42] and the addition of several MAMPs specific experiments sampling at early time-points would be required to pass this threshold for a distinct *CBP60g* analysis. The presence of *SARD1* and absence of *CBP60g* in the *SID2* cluster with the more stringent correlation threshold of experiment #1 (Figure 4a) imply that the combined dataset may be weighted towards later timepoints in infection, understandably since these have higher probability of uncovering large scale differential expression in relatively costly gene expression profiling studies. CBP60g has recently been implicated in mediating responses to abiotic stress such as drought [43]. Whilst many abiotic stress studies were included few passed the threshold for *CBP60g* or *SARD1* correlation with *SID2*. Closer inspection of two well-characterised studies of drought and osmotic stresses (NASCARRAYS – 139 and 141) revealed strong up-regulation of *CBP60g* in response to stress with only weak *SID2* response and hence low correlation. A similar pattern was observed in several other experiments with an abiotic stress treatment. Preliminary analysis revealed *CBP60g* to be co-expressed with a subset of the genes from the 518 used in experiment #2 with *SARD1* co-expressed with only one gene across this dataset (Additional file 7: Figure S3). Once more information has been uncovered concerning the role of CBP60g in mediating abiotic stress responses, revisiting such a co-expression analysis may prove fruitful.

Potentially the most exciting finding from the co-expression analysis of *CBP60g* and/or *SARD1* and *SID2* has been the two calmodulin-like genes in the core *SID2* cluster from experiment #1. Their strength of correlation combined with the frequency of GAAATT motifs in both *CML46* and *CML47* promoters and their potential for physically interacting with CBP60g makes them ideal candidates for further investigation. While the pathogen associated induction of *CML47* was dependent on CBP60g and SARD1 there was but a weak and insignificant impact on *CML46.* A further distinction between the two is that in the main analysis *CBP60g* and *CML46* have a Spearman correlation co-efficient of 0.84 and 0.74 in the abiotic stress data set used in Additional file 7: Figure S3, whereas between *CBP60g* and *CML47* the value falls from 0.76 to 0.31 indicating a potential abiotic stress specific role for CML46. The up-regulation of *CML45* (unmonitored by the ATH1 microarray) in *cbp60g sard1* mutants was also surprising and points to potentially complex interplay of feedback loops in controlling the expression of these putative CBP60g interactors.

Zooming out of the core SID2 cluster by relaxing the co-expression threshold in experiment #2 revealed several more putative targets for CBP60g and SARD1 regulation. Signalling components such as the kinases in this cluster provide key targets for investigating pathogen susceptibility in knock-out mutants as these may lie upstream of key defence responses. Cluster 2 already includes several genes known to confer resistance to infection. However, several of these (*PAD4, EDS1, WRKY46*) lie upstream of *CBP60g* and *SARD1*[7,20] and so while their presence in this cluster provides an interesting insight into the various feedback loops that govern their co-expression they will not explain the SID2-independent defence response. Two genes affecting pathogen resistance that may be downstream of *CBP60g* and *SARD1* are *SOBIR1* and *ADR1-L1*. *SOBIR1* is a negative regulator of defence responses [36] and so a poor candidate but the NB-LRR receptor *ADR1-L1* has been implicated as a positive regulator in the establishment of PTI, ETI and basal defence against virulent pathogens [35].

Outside the clusters containing *SID2,* cluster 17 of experiment #2 contains the greatest density of observed *cbp60g sard1* dependent genes with *PBS3* and *AIG1*, known from the custom array study, and *FMO1* and *CML47* confirmed by qPCR [20]. *FMO1* regulates *EDS1*-dependent, *SID2*-independent defence signalling, a process inhibited by *NUDT7* the homolog of which, *NUDT5*, is present in this cluster [44].

Of the genes whose promoters have abundant GAAATT motifs and which cluster within GAAATT enriched regulons there are perhaps four ways to account for those whose expression are not *CBP60g / SARD1* dependent. Firstly, since GAAATT is a relatively abundant sequence within the promoters of Arabidopsis genes with an average of close to two motifs per 1500 bp promoter the chance occurrence of two genes with several motifs within a small cluster of, say, five genes will easily produce false positives. It is however difficult to say how large a cluster should be to be worthy of investigation since at the higher stringency of experiment #1 clusters we know to be of interest can be relatively small. A second possibility is that, for CBP60g / SARD1 function, binding to the GAAATT motif is necessary but not sufficient and additional transcription factors and their binding sites are required for cooperative activation. The heatmap in Figure 8 implies that WRKY28 is not essential for such activation. However, the *SID2* cluster and several other GAAATT rich clusters are enriched with the consensus W-box element for WRKY binding. Third, for some clusters although GAAATT enrichment may accurately imply CBP60g SARD1 binding, another transcription factor may exert dominant control over induction in response to biotic challenge. Thus the influence of CBP60g and SARD1 may only be observed when this factor is absent. Finally, there are six other members of the CBP60 family with as yet no demonstrated DNA binding potential but in some instances moderate induction in response to biotic stresses which may potentially interact with some motif similar to the one defined for CBP60g and SARD1.

The role of CBP60g and SARD1 in repressing the expression of genes with multiple GAAATT motifs and co-expression with other GAAATT enriched genes was surprising given our previous finding of a significant under-representation of GAAATTT motifs in the promoters of genes up-regulated in the *cbp60g sard1* double mutant [20]. However, such complexity is not without precedence. The calmodulin-regulated transcription factor *SR1* has been shown to positively regulate *CBF2* but negatively regulate *EDS1*[45,46]. The complexity of Ca^2+^ mediated control over SA-mediated defence signalling is further underlined by the observation that SR1 inhibits expression of two important positive regulators upstream of *SID2*, *EDS1* and *NDR1*, but also inhibits the negative regulator of *SID2* expression, EIN3 [45,47]. Interestingly, numerous clusters appear to be enriched for SR1 binding sites with some overlapping GAAATT enrichment (Additional file 6: Table S4). Cluster 8 revealed several genes up-regulated in the absence of CBP60g and SARD1 and multiple members of this cluster act as inhibitors of defence responses (*BON1, BAP1, GILP, NUDT7*) [44,48,49]. Furthermore, PBP1 mediates auxin signalling, a potential inhibitor of SA-mediated defence signalling [50,51]. Another important defence component in this cluster is *MPK11*. While *MPK11* is significantly down-regulated in *cbp60g sard1* plants 24 hpi with *Pma* ES4326, it may be that the moderate increase in the mock inoculated or basal state is more important since MPK11 is activated within minutes of biotic challenge and steady-state transcript levels may be important [41]. Another feature of this cluster is the significant over-abundance of calcium binding proteins including BON1, BAP1, PBP1, CML37 and At3g10830, along with the calmodulin dependent kinase CPK substrate CZF [52]. These features combine to make this particular regulon an intriguing target for further investigation.

The process involved in the identification of WRKY28 as a regulator of *SID2* led us to expect *WRKY28* would form some part of the *SID2* cluster. The clear evidence that *WRKY28’s* association with *SID2* exists as a subset of the conditions under which there is a strong and significant association between *CBP60g* and / or *SARD1* and *SID2* implies some specific role for WRKY28. However, there was no clear trend in the conditions under which *WRKY28* was correlated with *SID2*. One trend appearing to emerge from the conditions where *SID2* was correlated with *CBP60g* and/or *SARD1* but not *WRKY28* were experiments in which the treatment was an exogenous application of SA or some analogue of SA such as benzothiadiazole (BTH), 3,5-dichloroanthranilic acid (DCA) or 2,6-dichloroisonicotinic acid (INA). For example, in experiments with exogenous SA application, NASCARRAYS192 and NASCARRAYS365, the *WRKY28:SID2* correlation is −0.17 and 0.18 while the *SARD1:SID2* correlation is 0.71 and 0.68. In experiments with BTH application, GSE9955 and NASCARRAYS392, *WRKY28:SID2* correlation scores are −0.48 and −0.03 while *SARD1:SID2* scores 0.86 and 0.89. In experiment GSE13833 [53], where DCA and INA are applied, the scores are 0.16 and 0.85 respectively. While these experiments are not sufficient to construct a stable co-expression matrix they suggest a role for *SARD1* in amplifying an existing SA-mediated signal through a feed-forward loop that is independent of *WRKY28*.

## Conclusions

Co-expression analysis has facilitated the identification of an SA-mediated defence signalling regulon at two different degrees of resolution. The promoters of these genes are enriched for a fragment of an oligomer demonstrated to bind to CBP60g and SARD1, indicating that some members of these clusters are likely targets for regulation by CBP60g and SARD1. Other putative targets have been identified in separate clusters and intriguingly some genes downstream of GAAATT-abundant promoters have been shown to be repressed by CBP60g and SARD1, indicating a potentially complex role in the control of defence gene expression responses. This co-expression analysis has also shed light on the relationship between *WRKY28* and *SID2* which may allow fine-tuning of regulatory models.

## Methods

### Plant growth conditions and pathogen cultures

*Arabidopsis thaliana* accession Col-0 was used as the wildtype control and *cbp60g-1* (SALK_023199) [18] and *sard1-2* (SALK_052422) [19] as the mutant lines. Plants were grown on autoclaved BM2 Germinating Mix (Berger) in a growth chamber with a 12 h photoperiod at 22°C with 75% humidity. Plants were grown for 4–5 weeks before bacterial infection or MAMPs treatment. *Pma* ES4326 cultures were grown overnight in King’s B medium with 100 μg ml^-1^ streptomycin at room temperature. Cultures were centrifuged, washed and resuspended in 5 mM MgSO_4_ to a density of OD_600_=0.01. Flg22 peptide was purchased from EZBiolab and prepared to 1 μM. MAMPs and bacterial inoculations were made with a needless syringe; mock treatments were 5 mM MgSO_4_.

### Quantitative RT-PCR analysis

RNA was purified using Trizol (Invitrogen) and treated with DNaseI (NEB). Quantitative RT-PCR experiments were performed using a Lightcycler 480 Real-Time PCR system (Roche). 24 ng of total RNA was used for each 10 μl reaction with the SuperScript III Platinum SYBR Green One-Step quantitative RT-PCR kit (Invitrogen) according to the protocols of the manufacturers. The thermal cycling program was 50°C for 10 min, 95°C for 10 min followed by 40 cycles of 95°C for 15 sec and 60°C for 1 min. For each reaction amplification curve the crossing point (Cp) was calculated using the 2^nd^ derivative max method provided with the Lightcycler software. Each reaction was run with two technical replicates and the Cp values for these replicates were averaged. Either four or five independent biological replicates were included for each gene and *Actin2* (*At3g18780*) was used as the internal reference. The following model was fit to the Cp value data using the lme function in the nlme package in the R environment: *Cp*_*gytr*_ = *GYT*_*gyt*_+*R*_*r*_+*ε*_*gytr*_, where *GYT* is the gene:genotype:treatment interaction as a fixed effect, and *R* (replicate) and *ε* (residual) are random effects. Once modelled, the mean estimate of the gene:genotype:treatment interaction was used as the Cp value and relative log_2_ expression values were obtained by subtracting Cp values of the *Actin2* gene. For two-tailed *t*-tests, the standard error appropriate for each comparison was calculated using the variance and covariance values obtained from the model fitting. A full list of the primers used can be found in Additional file 8: Table S5.

### Microarray data analysis

Affymetrix ATH1 whole genome microarray datasets were downloaded in the form of .CEL files from the following data repositories: NASCArrays (http://affymetrix.arabidopsis.info/narrays/experimentbrowse.pl); NCBI Gene Expression Omnibus (http://www.ncbi.nlm.nih.gov/geo/); EBI ArrayExpress (http://www.ebi.ac.uk/arrayexpress/); Ausubel lab IMDS (http://ausubellab.mgh.harvard.edu/imds/). A list of the experiments included in this study can be found in Additional file 9: Table S6. Data quality of the individual arrays were assessed for each experiment using the affyQCReport package, part of the Bioconductor suite of programs within the R environment [54]. Outlying arrays which appeared to distort the normalisation of the experiment were discarded. Each experiment was pre-processed and quantile normalised using the RMA algorithm as implemented by the justRMA function of the gcrma R package [55]. For each experiment the Spearman rank correlation coefficient, and corresponding p-value for this test, between probesets representing *SID2* (262177_at) and *CBP60g* (246821_at) or *SARD1* (260046_at, 260068_at) were determined using the cor and cor.test R functions. Due to prior misannotation of *SARD1*, two probesets report *SARD1* expression on the ATH1 chip but analysis of selected experiments revealed that the 260046_at probeset was the more reliable and sensitive of the two and is used in Figure 2. Correlation was scored using the Spearman rank correlation coefficient on the presumption that a non-parametric test would be more robust in the face of presumably widely varying data structures and that it would provide a more conservative measure of correlation, particularly in analyzing smaller datasets. Experiments were filtered based on the maximum significant correlation (Spearman correlation ≥ 0.7, p-value ≤ 0.05) of either *CBP60g:SID2* or *SARD1:SID2*.

### Co-expression analysis

For each of the selected microarray experiments the log_2_ expression values for each probeset were normalised such that the median value was set to 0. Datasets were merged and each probeset across all arrays was normalised such that the standard deviation was set to 1. 45 genes were identified as significantly differentially expressed in the custom mini-array experiments previously described (GEO: GSE18865 [20]) in *cbp60g*, *sard1* or *cbp60g sard1* plants relative to wildtype inoculated with either virulent *Pma* ES4326 or disarmed *Pto* DC3000 *hrcC*^-^ with at least 2 fold down-regulation and a false discovery rate of 0.05 or less. Genes co-expressed with these 45 seeds were selected based on the Spearman rank correlation coefficient with a threshold of 0.8 for experiment #1 and 0.7 for experiment #2. Correlation matrices were calculated for both experiments and connections between genes set at the 0.8 and 0.7 thresholds, respectively. DPClus was used to cluster both experiments with the following parameters: minimum cluster density of 0.75; CP threshold of 0.75; minimum cluster size of 3 for experiment #1 and 5 for experiment #2; allowing overlapping clusters to form [29]. Clusters were visualised using CYTOSCAPE [56].

For the abiotic stress co-expression network constructed for Additional file 7: Figure S3, 27 experiments comprising 501 arrays were selected based on the induction of *CBP60g* in response to an abiotic stress without strong correlation between *CBP60g* and *SID2* (≤0.5) (Additional file 9: Table S6). Genes co-expressed with either *CBP60g* or *SARD1* above a threshold of 0.7 across these experiments were included in the network.

For hierarchical clustering of array experiment correlation coefficients the CLUSTER program was used to organise experiments into self organising maps, subsequently complete linkage hierarchical cluster using an uncentered correlation metric was applied. Clustering was visualised using TREEVIEW [57].

### Promoter analysis

Promoter sequences were retrieved from the RSAT database (http://rsat.ulb.ac.be/) with fixed 1500 bp sequences upstream of the transcription start site used in all analyses except Additional file 1: Table S1 [58]. 1500 bp promoters were chosen, despite the enrichment of GAATT motifs being more pronounced in 1000 bp promoters, in order to include the original *SID2* site identified by Zhang *et al*. [19]. Five gene promoter sequences were not available on the RSAT server as they are considered pseudogenes (*At4g13900, At1g21525, At4g14610, At3g48650, At2g24160*). Nevertheless, these genes were included, with the promoter sequences retrieved from the TAIR database (http://www.arabidopsis.org/) [59]. The over-representation of known promoter cis-elements and motifs was assessed using the POBO application (http://ekhidna.biocenter.helsinki.fi/poxo/pobo/pobo) [31]. For each set of promoters, 1000 pseudoclusters of a size equal to the cluster in question were generated both from within the genes in question and the Arabidopsis genomic background (clean). Statistical significance of the t-values generated by POBO was calculated using the linked Graphpad application for a two-tailed comparison. For selected clusters, motif finding algorithms were used to uncover additional potential cis-elements using the MEME and RSAT tool suites [60,61].

## Abbreviations

BTH: Benzothiadiazole; CaM: Calmodulin; Cp: Crossing point; DCA: 3,5-dichloroanthranilic acid; ETI: Effector triggered immunity; EMSA: Electrophoretic mobility shift assay; HPI: Hours post inoculation; INA: 2,6-dichloroisonicotinic acid; MAMP: Microbe associated molecular pattern; OG: Oligogalacturonide; *Pma*: *Pseudomonas syringae* pv *maculicola*; PTI: Pattern triggered immunity; SA: Salicylic acid; SAR: Systemic acquired resistance.

## Competing interests

The authors declare that they have no competing interests.

## Authors’ contributions

WT carried out all analyses and wrote the paper. JG provided helpful suggestions and edited the manuscript. Both authors read and approved the final manuscript.

## Supplementary Material

Additional file 1 **Table S1.** Significance of various motifs within promoters of genes clustered with *SID2* Motifs are derived from the 10mer oligo found to bind CBP60g and SARD1 *in vitro* [19]. POBO analyses of a cluster of 11 genes including *SID2* identified during pilot co-expression analysis are reported as t-values from two-tailed *t*-tests. Positive values indicate an enrichment of motifs compared to the genome background and negative values an under-representation with increasing magnitude indicating greater significance. Promoter regions were defined as starting at the transcription start site. Click here for file

Additional file 2 **Table S2.** Overview and cluster contents of co-expression experiment #1. Click here for file

Additional file 3 **Table S3.** Overview and cluster contents of co-expression experiment #2. Click here for file

Additional file 4 **Figure S1.** CBP60g and SARD1 exert antagonist effects on the expression of phylogenetically related calmodulin-like genes. qRT-PCR measurement of gene expression 24 hpi *Pma* ES4326 (OD_600_ =0.01). Data from five biological replicates were merged using a mixed linear model and the mean log_2_ ratio to *Actin2* expression plotted along with the standard error. Asterisks denote a significant differential expression between wildtype and the *cbp60g sard1* mutant with p-value ≤ 0.05 from a two-tailed *t*-test. Click here for file

Additional file 5 **Figure S2.** Distribution of selected motifs in the *SID2* regulon. Visualisation of the distribution of GAAATT and CCTN7TCC motifs throughout cluster 2 of experiment #2. Plot created using the feature map function of the RSAT suite of tools (http://rsat.ulb.ac.be/). Red ticks denote CCTN7TCC and blue ticks represent GAAATT motifs, ticks above the promoter line are in the sense orientation and ticks below the line antisense. There is a significant bias of the CCTN7TCC motif towards the 750 bp proximal to the transcription start site (p-value=0.005). Click here for file

Additional file 6 **Table S4.** POBO analysis of motif enrichment in the promoters of clusters from experiment #1 and experiment #2. POBO analysis of the abundance of assorted motifs in the 1500 bp promoter region of clustered genes. 1000 pseudoclusters of size matched to the given cluster were generated both from within the cluster and the genome background. Significance is recorded as t-values from two-tailed *t*-tests. Positive values indicate an enrichment of motifs compared to the genome background and negative values an under-representation with increasing magnitude indicating greater significance. Motifs are derived from various sources. (1) The 10mer oligo found to bind CBP60g and SARD1. (2) Known W-box consensus sequences involved in binding WRKY transcription factors. (3) A novel dyad motif identified in a cluster containing *SARD1* and *SID2*. (4) An uncharacterised motif enriched in several clusters containing *CBP60g*. (5) The binding site for the *SID2* repressor EIN3. (6) The core binding site for NAC transcription factors including the negative regulator of *SID2,* ANA019. (7) The binding site for SR1 (CAMTA3), the negative regulator of *EDS1* and other defence signalling components. Click here for file

Additional file 7 **Figure S3.***CBP60g* forms a *SID2* independent co-expression network in response to abiotic stress. A network was created from the genes co-expressed with *CBP60g* and *SARD1* across 27 selected abiotic stress microarray datasets where *CBP60g* was induced by stress but not strongly correlated with *SID2* expression. Edges represent a Spearman rank correlation coefficient of at least 0.7; the width is proportional to the correlation between two genes. Click here for file

Additional file 8 **Table S5.** Primer sequences used for qRT-PCR. Click here for file

Additional file 9 **Table S6.** Overview of microarray data used for co-expression analysis. For each experiment the Spearman rank correlation coefficient between *SID2* and *WRKY28*, *CBP60g* and the two *SARD1* probesets is followed by the p-values associated with these correlations. Experiments used for the abiotic stress co-expression network are marked. Click here for file
