# Supplementary material for: Co-expression analysis identifies putative targets for CBP60g and SARD1 regulation
Source: BMC Plant Biol. 2012 Nov 16;12:216. doi: 10.1186/1471-2229-12-216 (PMC3511238; doi:10.1186/1471-2229-12-216)
Supplement: Additional file 1 — Table S1. Significance of various motifs within promoters of genes clustered with SID2 Motifs are derived from the 10mer oligo found to bind CBP60g and SARD1 in vitro [19]. POBO analyses of a cluster of 11 genes including SID2 identified during pilot co-expression analysis are reported as t-values from two-tailed t-tests. Positive values indicate an enrichment of motifs compared to the genome background and negative values an under-representation with increasing magnitude indicating greater significance. Promoter regions were defined as starting at the transcription start site. [file 1471-2229-12-216-S1.docx]

| **Supplemental Table 1: Significance of various motifs within promoters of genes clustered with *SID2*** | | | | | | |
| --- | --- | --- | --- | --- | --- | --- |
| Motif | **Promoter length** | | | | | |
|  | **3kb** | **2.5kb** | **2kb** | **1.5kb** | **1kb** | **0.5kb** |
| GAAATTTTGG | 20.95 | 24.58 | 24.26 | 25.69 | - | - |
| AAATTTTGG | 22.38 | 27.15 | 33.98 | 41.02 | 11.77 | 22.40 |
| GAAATTTTG | 33.83 | 37.08 | 27.90 | 31.64 | 34.10 | 41.12 |
| GAAATTTT | 24.39 | 35.21 | 26.68 | 34.38 | 37.40 | 41.13 |
| AAATTTTG | 32.45 | 27.25 | 11.89 | 24.15 | 14.15 | 27.68 |
| AATTTTGG | 18.00 | 25.18 | 37.34 | 47.53 | 21.21 | 25.81 |
| GAAATTT | 61.18 | 66.23 | 56.17 | 73.68 | 78.88 | 71.11 |
| AAATTTT | 48.45 | 51.78 | 16.21 | 26.43 | 8.62 | 24.25 |
| AATTTTG | 24.81 | 22.71 | 16.89 | 35.50 | 18.75 | 26.70 |
| ATTTTGG | -10.21 | 44.13 | 31.98 | 32.86 | 11.93 | -5.29 |
| GAAATT | 63.96 | 55.46 | 59.65 | **78.24** | **85.20** | 80.18 |
| AAATTT | 52.47 | 49.94 | 31.70 | 35.19 | 29.82 | 45.05 |
| AATTTT | 53.75 | 50.89 | 31.96 | 41.29 | 37.39 | 19.25 |
| ATTTTG | 56.82 | 33.05 | 27.54 | 21.05 | -5.04 | -25.34 |
| TTTTGG | 20.00 | 25.66 | 24.78 | 31.98 | 21.53 | 23.21 |
