# Supplementary figures and images for: Co-expression analysis identifies putative targets for CBP60g and SARD1 regulation
Source: BMC Plant Biol. 2012 Nov 16;12:216. doi: 10.1186/1471-2229-12-216 (PMC3511238; doi:10.1186/1471-2229-12-216)

## Slide 1
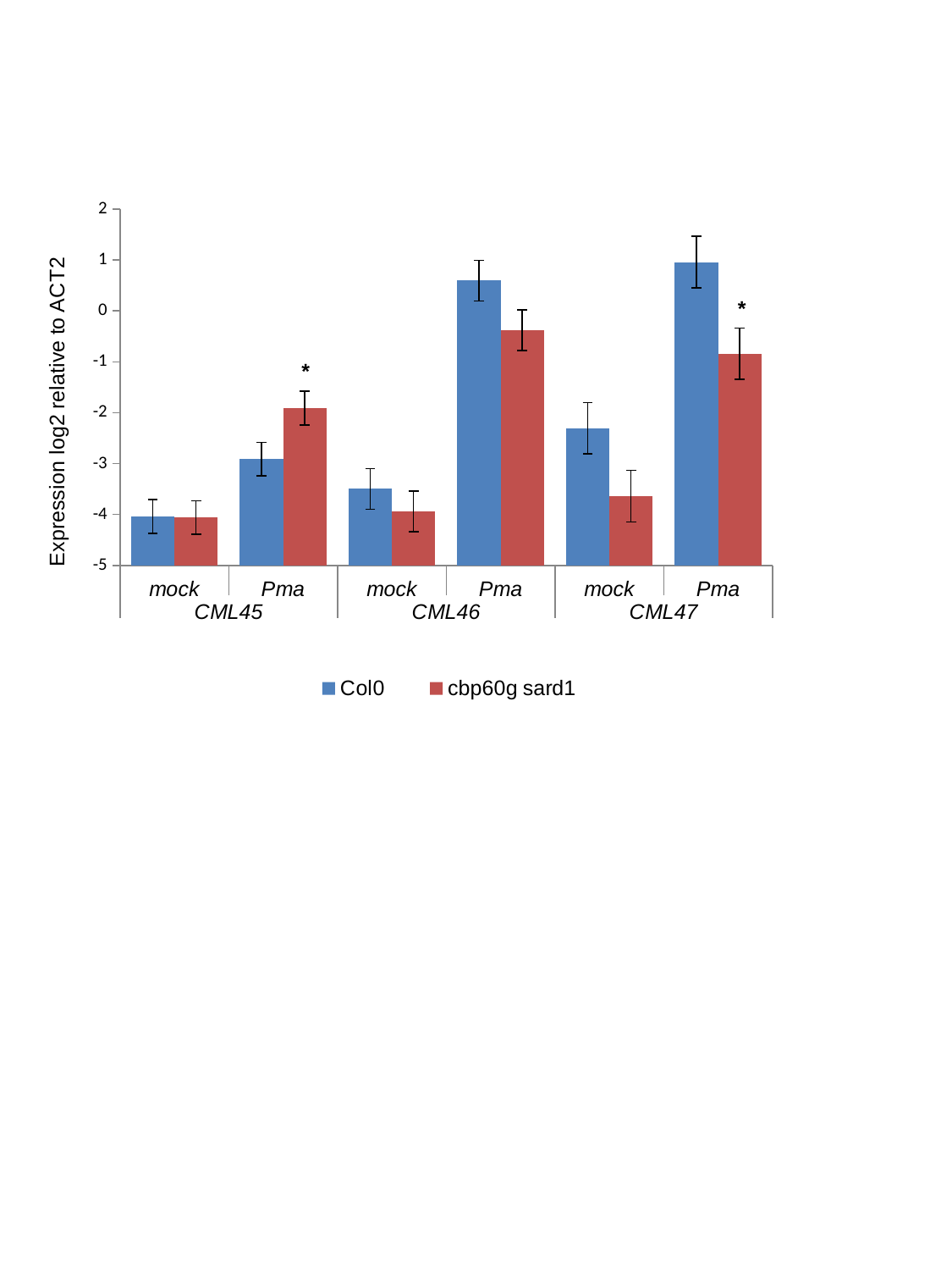

### Chart
| Category | Col0 | cbp60g sard1 |
|---|---|---|
| mock | -4.03857142857143 | -4.0600000000000085 |
| Pma | -2.9121428571428467 | -1.9092857142857242 |
| mock | -3.4960000000000178 | -3.941 |
| Pma | 0.594999999999999 | -0.37900000000000184 |
| mock | -2.3059999999999867 | -3.638 |
| Pma | 0.9600000000000046 | -0.8420000000000026 |*
*

Supplement: Additional file 4 — Figure S1. CBP60g and SARD1 exert antagonist effects on the expression of phylogenetically related calmodulin-like genes. qRT-PCR measurement of gene expression 24 hpi Pma ES4326 (OD600 =0.01). Data from five biological replicates were merged using a mixed linear model and the mean log2 ratio to Actin2 expression plotted along with the standard error. Asterisks denote a significant differential expression between wildtype and the cbp60g sard1 mutant with p-value ≤ 0.05 from a two-tailed t-test. [file 1471-2229-12-216-S4.pptx]

## Slide 1
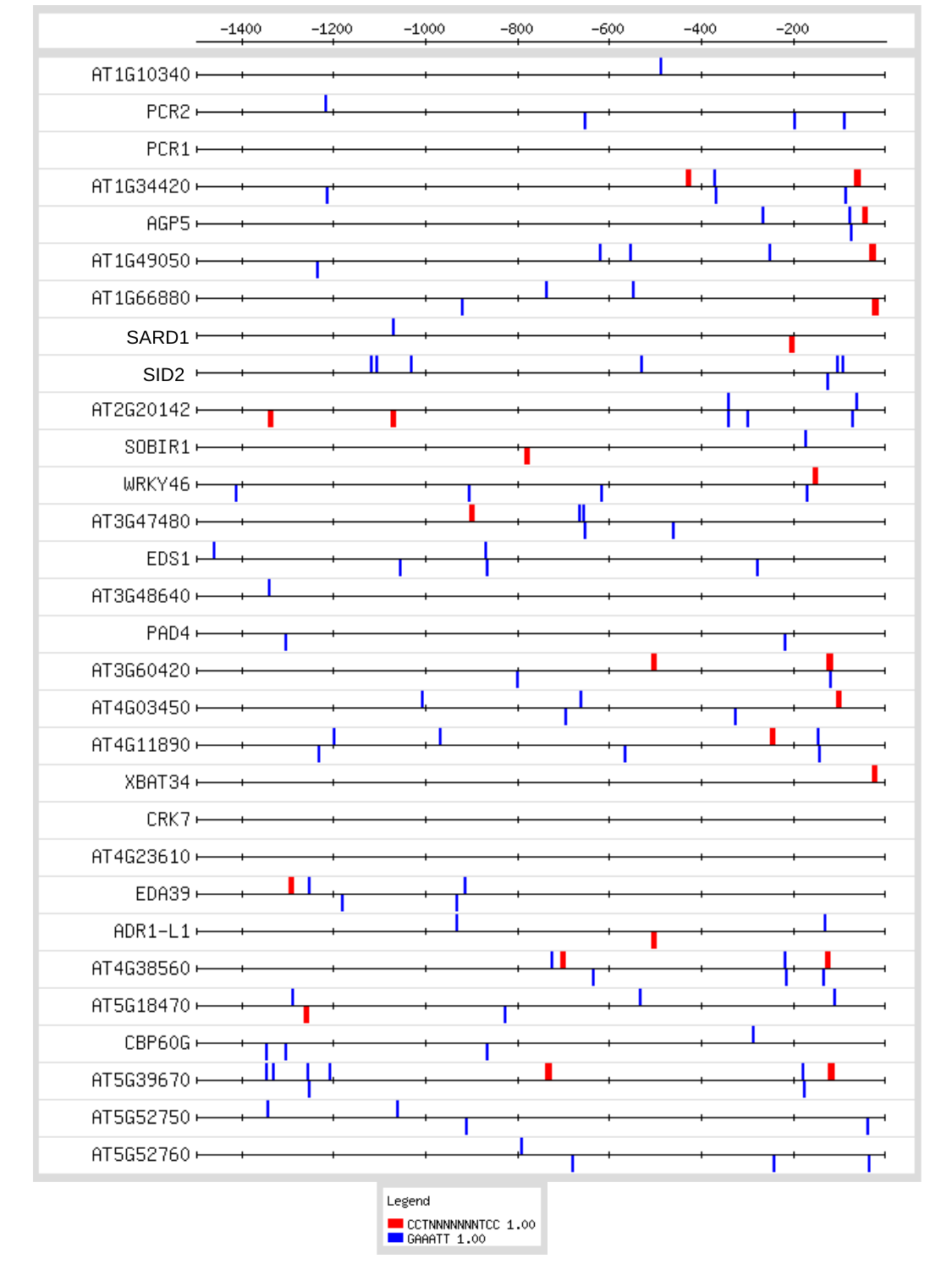

SARD1
SID2

Supplement: Additional file 5 — Figure S2. Distribution of selected motifs in the SID2 regulon. Visualisation of the distribution of GAAATT and CCTN7TCC motifs throughout cluster 2 of experiment #2. Plot created using the feature map function of the RSAT suite of tools (http://rsat.ulb.ac.be/). Red ticks denote CCTN7TCC and blue ticks represent GAAATT motifs, ticks above the promoter line are in the sense orientation and ticks below the line antisense. There is a significant bias of the CCTN7TCC motif towards the 750 bp proximal to the transcription start site (p-value=0.005). [file 1471-2229-12-216-S5.pptx]

## Slide 1
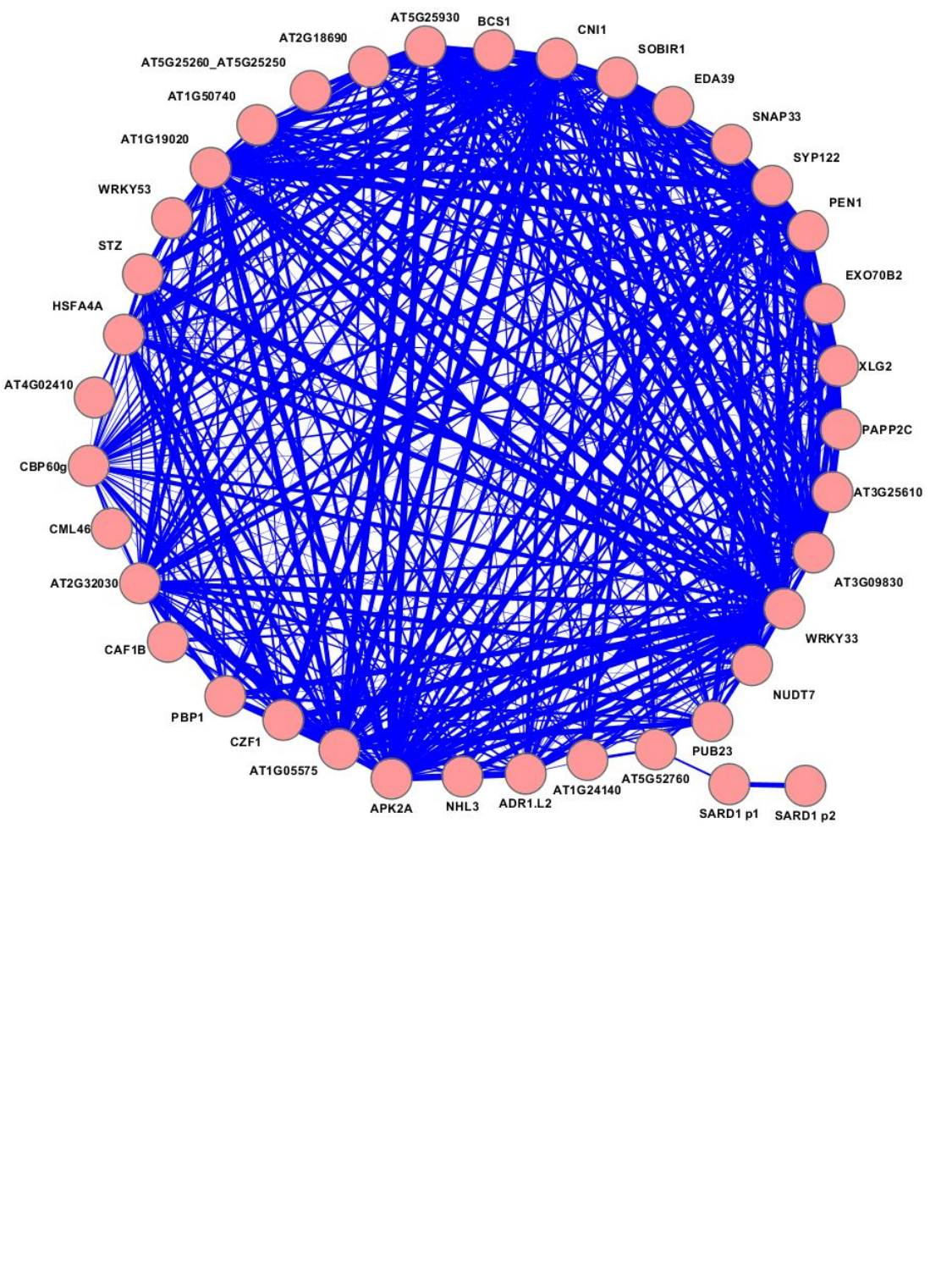

Supplement: Additional file 7 — Figure S3.CBP60g forms a SID2 independent co-expression network in response to abiotic stress. A network was created from the genes co-expressed with CBP60g and SARD1 across 27 selected abiotic stress microarray datasets where CBP60g was induced by stress but not strongly correlated with SID2 expression. Edges represent a Spearman rank correlation coefficient of at least 0.7; the width is proportional to the correlation between two genes. [file 1471-2229-12-216-S7.pptx]
